# Intranasal delivery of inactivated PRRSV loaded cationic nanoparticles coupled with enterotoxin subunit B induces PRRSV-specific immune responses in pigs

**DOI:** 10.1038/s41598-022-07680-9

**Published:** 2022-03-08

**Authors:** Puwich Chaikhumwang, Adthakorn Madapong, Kepalee Saeng-chuto, Dachrit Nilubol, Angkana Tantituvanont

**Affiliations:** 1 Division of Pharmaceutical Sciences, Department of Pharmaceutical Care, Faculty of Pharmaceutical Sciences, University of Phayao, Phayao, 56000 Thailand; 2 Swine Viral Evolution and Vaccine Development Research Unit, Department of Veterinary Microbiology, Faculty of Veterinary Science, Chulalongkorn University, Bangkok, 10330 Thailand; 3 Department of Pharmaceutics and Industrial Pharmacy, Faculty of Pharmaceutical Sciences, Chulalongkorn University, Bangkok, 10330 Thailand

**Keywords:** Immunology, Nanoscience and technology

## Abstract

This study was conducted to evaluate the induction of systemic and mucosal immune responses and protective efficacy following the intranasal administration of inactivated porcine reproductive and respiratory syndrome virus (PRRSV) loaded in polylactic acid (PLA) nanoparticles coupled with heat-labile enterotoxin subunit B (LTB) and dimethyldioctadecylammonium bromide (DDA). Here, 42- to 3-week-old PRRSV-free pigs were randomly allocated into 7 groups of 6 pigs each. Two groups represented the negative (nonvaccinated pigs/nonchallenged pigs, NoVacNoChal) and challenge (nonvaccinated/challenged, NoVacChal) controls. The pigs in the other 5 groups, namely, PLA nanoparticles/challenged (blank NPs), LTB-DDA coupled with PLA nanoparticles/challenged (adjuvant-blank NPs), PLA nanoparticles-encapsulating inactivated PRRSV/challenged (KNPs), LTB-DDA coupled with PLA nanoparticles loaded with inactivated PRRSV/challenged pigs (adjuvant-KNPs) and inactivated PRRSV/challenged pigs (inactivated PRRSV), were intranasally vaccinated with previously described vaccines at 0, 7 and 14 days post-vaccination (DPV). Serum and nasal swab samples were collected weekly and assayed by ELISA to detect the presence of IgG and IgA, respectively. Viral neutralizing titer (VNT) in sera, IFN-γ-producing cells and IL-10 secretion in stimulated peripheral blood mononuclear cells (PBMCs) were also measured. The pigs were intranasally challenged with PRRSV-2 at 28 DPV and necropsied at 35 DPV, and then macro- and microscopic lung lesions were evaluated. The results demonstrated that following vaccination, adjuvant-KNP-vaccinated pigs had significantly higher levels of IFN-γ-producing cells, VNT and IgG in sera, and IgA in nasal swab samples and significantly lower IL-10 levels than the other vaccinated groups. Following challenge, the adjuvant-KNP-vaccinated pigs had significantly lower PRRSV RNA and macro- and microscopic lung lesions than the other vaccinated groups. In conclusion, the results of the study demonstrated that adjuvant-KNPs are effective in eliciting immune responses against PRRSV and protecting against PRRSV infections over KNPs and inactivated PRRSV and can be used as an adjuvant for intranasal PRRSV vaccines.

## Introduction

Porcine reproductive and respiratory syndrome (PRRS) is an economically devastating disease in pigs characterized by reproductive failure and respiratory distress^1,2^. PRRS virus (PRRSV), an enveloped, positive-sense, single-stranded RNA virus belonging to the genus *Betaarterivius*, subfamily *Variarterivirinae*, family *Arteriviridae*, and order *Nidoviralase,* is the causative agent^3^. Presently, PRRSV has been classified into two genetically distinct species: *Betaarterivirus suid 1* (former PRRSV-1, European type) and *Betaarterivirus suid 2* (former PRRSV-2, American type)^4–6^. PRRSV-1 has predominantly been observed in European countries but has further evolved into 4 subtypes^7^. Meanwhile, PRRSV-2 has been dominant on the North American continent^1^ and has further evolved into 9 distinct lineages^8^. On the Asian continent, the coexistence of both genotypes has been reported in several countries^9,10^, including Thailand. Initially, the prevalence of PRRSV-1 and PRRSV-2 in Thailand was 66.42% and 33.58%, respectively. However, the proportion of these genotypes has continuously changed in favor of PRRSV-2 virus due to the intensive use of modified live PRRSV (MLV) vaccine, particularly the PRRSV-2 genotype. This has resulted in the predomination of PRRSV-2^11^, in which PRRSV-2 MLV is mainly used. In addition to coinfection with both PRRSV types, previous studies have shown that highly pathogenic PRRSV (HP-PRRSV) is a predominant population of PRRSV-2 on the Asian continent^9,11^. These scenarios make the prevention and control of PRRS on the Asian continent more difficult than that in other regions.

Vaccination has been used as part of a PRRSV control program with varying degrees of success. At present, several types of PRRSV vaccines are commercially available, including MLV, inactivated and subunit vaccines. However, the effectiveness of these vaccines remains unsatisfactory^12^. Contributing factors that affect vaccine efficacy include the quasi-species nature of PRRSV, which has resulted in genetic heterogeneity. Although homologous protection is complete, heterologous protection has varied from partial to none^13,14^. MLV has been widely used. However, it might be unsafe in susceptible pigs due to the risk of mutation of the virus used in the vaccine^15^. In addition, MLV confers complete protection against homologous viruses but not heterologous viruses^16,17^. The risk of virulence reversion and shedding by attenuated strains also limits MLV use^15^. Subunit PRRSV vaccines provide immune protection against PRRSV, although they have been reported to have some drawbacks, such as the exacerbation of respiratory disease in pigs following challenge with PRRSV after vaccination^12^ and the more complex manufacturing process that is required compared to that used for other vaccines^18^. Inactivated PRRSV vaccines are regarded as safe, but they have poor immunogenicity^19^.

An additional contributing factor could be the route of vaccine administration. Commercially available vaccines are recommended to be administered through an intramuscular route. This route of vaccination results in the induction of systemic immune responses but not mucosal immune protection^20,21^. PRRS is a viral infectious disease of the respiratory tract in pigs. The primary target cells of PRRSV are pulmonary alveolar macrophages, and the disease is initiated primarily in the lungs. Administration through a nasal vaccine is a promising option for the prevention and control of the disease. The presence of numerous microvilli covering the nasal epithelium provides a large area for vaccine absorption and allows a small dose of antigen to stimulate the immune system^22^. Furthermore, nasal vaccination is reportedly capable of inducing both mucosal and systemic immunity^23^. However, intranasal vaccination has some limitations, including rapid clearance, which leads to an insufficient uptake of antigen, and the lack of compatible mucosal adjuvants^23^. These limitations contribute to the failure of immune stimulation through the nasal route using inactivated antigens alone. To overcome these problems, nanoparticle-based vaccines and immunomodulating adjuvants, such as poly(lactic-co-glycolic acid) (PLGA), have been shown to successfully induce both systemic and mucosal immune responses when administered intranasally^24–26^.

Previously, we developed polylactic acid (PLA) nanoparticle-coupled adjuvants, namely, dimethyldioctadecylammonium bromide (DDA) and heat-labile enterotoxin subunit B (LTB)^27^. PLA nanoparticles coupled with DDA and LTB improved the transport of fluorescein isothiocyanate-labeled bovine serum albumin (BSA-FITC) across M cells and facilitated the uptake of BSA-FITC into porcine alveolar macrophages (PAMs) without causing toxicity in the cells. DDA, a cationic lipid, is utilized to create a positive charge on the surface of PLA nanoparticles, which demonstrates the ability to deliver antigens to enhance the uptake and presentation of antigens to antigen-presenting cells (APCs) and induce both mucosal IgA and systemic IgG compared to other cationic lipids^28,29^. However, cationic PLA nanoparticles were retained in the M cells, which resulted in a decrease in the likelihood of being engulfed by immune cells to further induce an immune response. To facilitate transcytosis and avoid particle retention in M cells, LTB, a mucosal adjuvant^30^, was used for coupling on the surface of cationic PLA nanoparticles, which promoted the function of M cells in the transcytosis of particles at the apical site and released them to the subepithelium where immune cells could process them further^31^.

Cationic PLA nanoparticles qualify as a mucoadhesive vaccine delivery system^32^ and are more effective in improving local and systemic immune responses than anionic PLA nanoparticles following intranasal administration^33^. The application of PLA nanoparticles coupled with DDA and LTB as an intranasal vaccine delivery for inactivated PRRSV might thereby improve the efficacy of inactivated PRRSV to effectively induce systemic and mucosal immune responses against PRRSV. Therefore, the present study was conducted to investigate the induction of systemic and mucosal immune responses and protective efficacy following the intranasal administration of PLA nanoparticles coupled with LTB and DDA and loaded with inactivated PRRSV.

## Materials and methods

### Cells culture and PRRSV

MARC-145 and 3D4/2 cells (porcine alveolar macrophages, PAMs) were purchased from the American Type Culture Collection (ATCC) (VA, USA) and used in the investigation. MARC-145 cells were cultured in minimal essential medium (MEM) (Thermo Fisher Scientific, MA, USA) with 5% fetal bovine serum (FBS) (Life Technologies, MD, USA) and antibiotic–antimycotic solution (Gibco Invitrogen Corporation, CA, USA). 3D4/2 cells are continuous porcine cell lines developed from alveolar macrophages in which their characterization and virus susceptibility have been reported elsewhere^34^. For propagation, the 3D4/2 cells were cultured in advanced RPMI 1640 media supplemented with 4.5 g/L D-(+)-glucose (Sigma–Aldrich, MO, USA), 10% FBS, antibiotic–antimycotic solution, and GlutaMAX. Cells were cultured and maintained in tissue culture flasks at 37 °C in a humidified environment of 5% CO_2_.

### PRRSV propagation and inactivation

PRRSV type 2 (S1/17 MA2-2 0117 ORF5US) were isolated from swine farms in Thailand experiencing PRRS virus outbreaks. The viruses were propagated in MARC-145 cells at 0.01 multiplicity of infection (MOI). MARC-145 cells were harvested when exhibiting 80% cytopathic effect (CPE) and supernatant was collected by centrifugation at 2500 rpm under 4 °C for 15 min. The PRRSV-containing supernatant was then overlaid onto 20% sucrose and ultracentrifuged at 35,000 rounds per minute (rpm) for 3 h to pellet the virus at 4 °C. Viral pellets were then resuspended in sterile PBS and inactivated with UV radiation dose of 1000 mJ/cm^2^ for 10 min, using a UV generator (Hoenle UV Technology, Munich, Germany). The inactivated virus was stored at − 80 °C until used.

### PRRSV complete inactivation analysis

To determine whether the virus was totally inactivated, MARC-145 cells were inoculated with 1 mL of each inactivated viral suspension. The cells were cultured for one week at 37 °C in a humidified environment with 5% CO_2_. MARC-145 cells were inoculated with 1 mL of noninactivated virus and mock medium as positive and negative controls, respectively. CPE was observed to identify infected cells.

### Total PRRSV protein determination

The total PRRSV protein content was determined using a bicinchoninic acid (BCA) assay kit (Sigma-Aldrich, MO, USA) by the help of series of bovine serum albumin (BSA) standard prepared in phosphate-buffered saline (PBS). The inactivated PRRSV was kept at − 80 °C until use.

### Formulation

#### Preparation of polylactic acid (PLA) nanoparticles loaded with inactivated PRRSV

PLA nanoparticles loaded with inactivated PRRSV (KNPs) were prepared according to our previous study using the double emulsion evaporation technique^27^. Briefly, inactivated virus pellets equivalent to 5 mg total viral protein content were added to 5% Resomer 202H poly-(D,L-lactide) or PLA (MW 10,000–18,000 gmol^−1^) (Evonik Nutrition & Care GmbH, Essen, Germany) in dichloromethane (DCM) solution (EMSURE, Merk, Germany). The mixture was sonicated at 30% amplitude for 1 min using a SON-1 VCX750 probe sonicator (Sonics & Materials, Inc., CT, USA) to form a primary emulsion. The emulsion was then added to 5% polyvinyl alcohol solution (Sigma–Aldrich, MO, USA) and sonicated at 30% amplitude for 2 min to generate a water-in-oil-in-water (w/o/w) double emulsion. The double emulsion was stirred overnight at ambient temperature to obtain nanoparticles encapsulated with inactivated PRRSV. The nanoparticles were washed three times with deionized water, ultracentrifuged (Beckman Coulter Inc., CA, USA) at 35,000 rpm at 4 °C for 30 min, freeze dried using a lyophilizer (Lyophilization system, Inc., NY, USA) and stored at 4 °C.

For the preparation of LTB-DDA coupled with PLA nanoparticles loaded with inactivated PRRSV (adjuvant-KNPs), an aliquot of inactivated virus pellets was emulsified with the mixture of PLA and DDA in DCM under the same conditions as previously described^27^. After that, ten milligrams of the freeze-dried nanoparticles were resuspended in 1 mL of PBS (pH 7.4) containing 2 μg of LTB (Sigma–Aldrich, MO, USA) and mixed for 10 min before use. PBS was used instead of inactivated virus pellets as a blank in the preparation of PLA nanoparticles (blank NPs) and LTB-DDA coupled with PLA nanoparticles (adjuvant-blank NPs). Both groups were served as nanoparticle controls.

### Characterization of the nanoparticles

#### Size, size distribution and zeta potential measurement

The particle size, size distribution and zeta potential of the freeze-dried nanoparticles were determined using a Zetasizer Nano ZS (Malvern, UK). Ten milligrams of freeze-dried nanoparticles were resuspended in 1 mL of deionized water and subjected to size analysis. All measurements were performed in triplicate at 25 °C.

#### Scanning electron microscope

The morphology of the freeze-dried nanoparticles was visualized using scanning electron microscopy (JEOL Ltd., Tokyo, Japan). The nanoparticles were mounted on adhesive tape and coated with gold-platinum under a vacuum using an ion coater (Blazers, Liechtenstein). The coated samples were examined under a microscope at 10 kV (JSM-6610) HV/LV with EDX and at a magnification of 15,000 ×.

#### Protein entrapment efficiency (EE) of nanoparticles

Ten milligrams of freeze-dried nanoparticles were degraded in 1 mL of 0.1 N NaOH for 1 h at ambient temperature with a constant stirring at 500 rpm. The mixture was ultracentrifuged at 35,000 rpm for 10 min. After that, the supernatant was collected to determine the total viral protein content using a BCA protein assay kit. The % EE was calculated by dividing the amount of encapsulated protein by the total amount of added protein as the following equation:

$$\% {\text{EE}} = \frac{{{\text{The}}\;{\text{amount}}\;{\text{of}}\;{\text{entrapped}}\;{\text{protein}}\;{\text{in}}\;{\text{nanoparticles}}}}{{{\text{The}}\;{\text{total}}\;{\text{amount}}\;{\text{of}}\;{\text{added}}\;{\text{protein}}}} \times 100.$$

The series of BSA samples with different concentrations in 0.1 N NaOH served as the reference standards.

#### Western blot analysis of PRRSV protein in nanoparticles

Freeze-dried nanoparticles were degraded for 24 h at room temperature in 1 mL of 0.1 N NaOH with continuous stirring at 500 rpm. The suspension was ultracentrifuged for 10 min at 35,000 rpm. The supernatant was then separated and quantified for the total viral protein content using a BCA assay kit. The sample was prepared in sodium dodecyl sulfate–polyacrylamide gel electrophoresis (SDS–PAGE) loading buffer (Bio–Rad, CN, USA) with equal amounts of viral protein and boiled for 5 min before loading onto a 15% acrylamide gel. SDS–PAGE was used to separate viral proteins according to the manufacturer's recommendations (Bio–Rad, CN, USA). Plus Protein WesternC Blotting Standards (Bio–Rad, CN, USA) were used as molecular-weight size markers. Viral proteins were transferred onto a nitrocellulose membrane for western blot detection using a Trans-Blot SD Semi-Dry Transfer Cell (Bio–Rad, CN, USA). The membranes were incubated for 1 h at room temperature in blocking buffer (5% skim milk in Tris-buffered saline and Tween 20; TBST). The viral proteins were detected using antibody in serum generated from a pig vaccinated with PRRS MLV, which was diluted in blocking buffer overnight at 4 °C. The membranes were washed twice with TBST and incubated with HRP-coupled secondary antibody in blocking buffer for 1 h at room temperature. The antigen–antibody complexes were examined using chemiluminescence substrate (SuperSignal West Pico, MA, USA).

### In vitro cell culture studies

#### 3-(4,5-Dimethylthiazol-2-yl)-2,5-diphenyltetrazolium bromide (MTT) cell viability assay

3D4/2 cells (PAMs) were seeded in 96-well plates at a concentration of 4.5 × 10^4^ cells/well and incubated with inactivated PRRSV, adjuvant-blank NPs, KNPs and adjuvant-KNPs at 37 °C under a 5% CO_2_ containing humidified atmosphere. After 24 h of incubation, 20 μL of MTT solution at a concentration of 1 mg/mL was added to the PAMs and incubated for another 4 h. The formazan crystals formed by the metabolically active cells were dissolved in 200 μL of dimethyl sulfoxide (DMSO) and quantified by measuring the absorbance at 540 nm using a CLARIOstar microplate reader (BMG LABTECH, Ortenberg, Germany). The percentage of cell viability was calculated by dividing the absorbance of the treated cells by that of the control cells used in each experiment. Untreated PAMs were used as the control cells.

#### Determination of interferon gamma (IFN-γ) production in PAMs

3D4/2 cells (PAMs) were incubated with inactivated PRRSV, adjuvant-blank NPs, KNPs and adjuvant-KNPs for 48 h at 37 °C in a humidified environment with 5% CO_2_. Untreated PAMs were used as the control. PAMs were then incubated with either MARC-145 cell lysate (negative control), phytohemagglutinin (Sigma–Aldrich, MO, USA) or homologous PRRSV at a 0.01 MOI. After incubation, a protein transport inhibitor (BD GolgiStop, BD Biosciences, CA, USA) was added to the PAMs 12 h before cell harvesting. The PAMs were subsequently fixed in fixation buffer (BD Pharmingen, CA, USA) and stained with biotin-conjugated anti-pig IFN-γ (BD Pharmingen, CA, USA) overnight at 4 °C, followed by incubation with streptavidin-conjugated PerCP (Thermo Fisher, MA, USA) for 1 h at 4 °C. The stained cells were washed and suspended in 2% paraformaldehyde prior to analysis using a flow cytometer (Beckman FC550, Beckman Coulter, CA, USA). The results are based on cells gating at least 20,000 cell events, as determined by a forward scatter versus side scatter graph.

### Animal studies

#### Experimental design

Forty-two, castrated male, crossbred (LRxLWxD) pigs of approximately 3 weeks of age were procured from a PRRSV negative herd. All pigs were tested for PRRSV by serological testing and PCR prior to shipment. Upon arrival, pigs were randomly allocated into 7 treatments groups of 6 pigs each. (1) Pigs in non-vaccinated/non-challenged (NoVacNoChal) and (2) non-vaccinated/challenged pigs (NoVacChal) groups were left as negative and challenge control. (3) Pig in PLA nanoparticles/challenged pigs (Blank NPs), (4) LTB-DDA coupled PLA nanoparticles/challenged pigs (Adjuvant-Blank NPs), (5) PLA nanoparticles loaded with inactivated PRRSV/challenged pigs (KNPs), (6) LTB-DDA coupled PLA nanoparticles loaded with inactivated PRRSV/challenged pigs (Adjuvant-KNPs) and (7) inactivated PRRSV/challenged pigs (Inactivated PRRSV) were intranasally vaccinated for 3 consecutive weeks (500 µL/nostril) at 0, 7 and 14 days post vaccination (DPV). The amount of PRRSV protein antigens in each vaccine dose was 1 mg. At 28 DPV, pigs in challenged groups were inoculated intranasally with PRRSV-2 (S1/17 MA2-2 0117) at 10^6^ TCID_50_ (1 mL/nostril). Pigs were euthanized at 7 days post challenge (DPC), 35 DPV. Macro- and microscopic lung lesions were examined. The animal studies were performed as previously described^35^.

All animal procedures were carried out in accordance with the requirements of the Guide for the Care and Use of Laboratory Animals of the National Research Council of Thailand according to protocols that were reviewed and authorized through the Chulalongkorn University Animal Care and Use Committee (protocol number 1731047). The animal study is reported in compliance with ARRIVE guidelines^36^.

#### Collection of blood, nasal swab samples and lung for analyses

Blood and nasal swab samples were collected on 0, 14, 28 and 35 DPV. Sera were separated and assayed for the presence of antibody using virus neutralization assay (VNA) and immunoglobulin (Ig) G enzyme-linked immunosorbent assay (ELISA). Nasal swab samples were obtained by deeply inserting swabs of cotton wool into nasal cavity and placed in sterile PBS. Swab samples were centrifuged and clarified supernatants were assayed for the presence of IgA antibody using ELISA. Lung tissues were collected at necropsy. Lung lysates were prepared and assayed for the presence of PRRSV RNA and antibody using PCR and VNA, respectively.

#### Preparation of lung lysates

Lung lysates were prepared with a slight modification according to a previously described protocol^37^. In brief, 5 g of lung tissue were minced into pieces and finely mashed using a mortar and pestle. Tissues were clarified by centrifugation at 2,500 rpm under 4 °C for 15 min. Supernatant was then collected, passed through 0.2 μm membrane filter and subjected for further determination of virus neutralizing titer (VNT).

#### Isolation of peripheral blood mononuclear cells (PBMCs)

PBMCs were isolated from heparinized blood sample by gradient density centrifugation (Lymphosep, Lymphocyte separation media, Biowest, USA) according to a previously described protocol^38^. The isolated PBMCs were resuspended in advanced RPMI-1640 (GIBCO, CA, USA) supplemented with 10% FBS, Antibiotic–Antimycotic Solution, Glutamax, 25 mM HEPES (Sigma-Aldrich, MO, USA) and 50 μM β-mercaptoethanol (Sigma Chemical Co., USA). Viability of PBMCs were determination using Trypan blue (Sigma-Aldrich, MO, USA) staining and subjected for further analyses including the measurement of IFN-γ and Interleukine-10 (IL-10).

#### Determination of virus neutralizing titer

To measure VNT in sera and lung lysates, VNA was performed. In brief, sera were heated inactivated at 56 °C for 30 min. To conduct VNA, sera were serially two-fold diluted and incubated with an equal volume of PRRSV (10^2^ TCID_50_). Following a 2 h incubation at 37 °C, 100 µL of the samples/virus mixes were transferred into a 96-well plate containing a confluence monolayer of MARC-145 cells. Cells were incubated at 37 °C for 48 h in a CO_2_ incubator prior evaluation of CPE through microscope. VNT in sera and lung lysates were determined by the highest dilution that inhibited CPE. Neutralization titers were expressed as the geometric mean titer (GMT) of the reciprocal of highest serum dilution that completely inhibit virus infection (no CPE).

#### Analysis of PRRSV-specific antibodies

The level of IgG in sera and IgA in nasal swab samples were determined using ELISA. Briefly, 96-well ELISA plates (Nunc MaxiSorp flat-bottom, Thermo Fisher Scientific, CPH, Denmark) were coated with whole PRRSV (10 µg/mL) in carbonate buffer (pH 9.6) and incubated overnight at 4 °C. ELISA plates were washed and treated with blocking buffer containing 1% BSA and 0.1% Tween 20 in PBS for 2 h at ambient temperature. Sera or nasal swabs samples were added and incubated for 2 h at ambient temperature. Plates were then washed with blocking buffer and incubated with either anti-pig IgA-horseradish peroxidase (HRP) or anti-pig IgG-HRP (Bio-Rad Laboratories, CA, USA) for 3 h at ambient temperature to detect IgG and IgA in serum and nasal swab samples, respectively. Following a wash, 3,3',5,5'-tetramethylbenzidine (TMB) (Sigma-Aldrich, MO, USA) was added. The reaction was stopped and the optical density was measured at 450 nm using an ELISA plate reader (AccuReader, Taipei, Taiwan). Data were analyzed as OD values which were subtracted from those of control wells.

#### Determination of lymphocytes producing IFN-γ

The percentage of PRRSV-specific lymphocytes producing IFN-γ after stimulation with homologous virus was evaluated using a flow cytometric technique. Briefly, 1 × 10^6^ PBMCs were seeded into a 96-well plate containing a mock suspension, PMA (25 ng/ml)/ionomycin (1 μM) (Sigma–Aldrich, MO, USA), and homologous PRRSV at 0.01 MOI and incubated for 96 h. Following incubation, a protein transport inhibitor (BD GolgiStop, BD Biosciences, CA, USA) was added 6 h prior to cell harvesting and staining. The labeled PBMCs were stained with mouse anti-porcine CD4-FITC antibody and mouse anti-porcine CD8-SPRD antibody (Southern Biotech, AL, USA) for 30 min at 4 °C. Subsequently, PBMCs were fixed and permeated with fixation/permeation buffer (BD Cytofix/Cytoperm Plus, CA, USA) for 45 min, washed and then incubated with mouse anti-porcine IFN-γ-PE antibody (BD Pharmingen, CA, USA) for 45 min at 4 °C in the dark. After washing, the stained cells were suspended in 1% FBS in PBS and analyzed by a flow cytometer with CellQuest software (BD FACSCalibur, Erembodegem, Belgium). The cells were gated into CD4^+^CD8^+^, CD4^+^CD8^−^ and CD4^−^CD8^+^ lymphocytes (Supplementary Fig. S1). The percentage of lymphocytes producing IFN-γ was analyzed based on lymphocyte gating, as determined by a forward scatter versus side scatter graph after the acquisition of at least 30,000 events.

#### Quantification of porcine IL-10 from PBMCs

Porcine IL-10 concentration was quantified in supernatants of stimulated PBMCs (1 × 10^6^ cells/well) cultured with homologous PRRSV (MOI of 0.01) using a porcine IL-10 ELISA kit (Quantikine ELISA Porcine IL-10, R&D System, Minneapolis, USA) according to the manufacturer’s instructions.

#### Quantification of PRRSV RNA copy number

PRRSV RNA in the lungs was evaluated by quantitative PRC (qPCR) after PRRSV challenge. Briefly, total RNA was extracted from lung samples (25 mg wet weight/pig) using a NucleoSpin RNA Virus tissue extraction kit (Macherey–Nagel, Germany) in accordance with the manufacturer’s protocol. The RNA quality was measured using a NanoDrop spectrophotometer (Colibri Spectrometer, Titerterk-Berthold, Germany) and converted to cDNA. The cDNA was used for qPCR. Primers specific for the ORF5 gene in PRRSV type 2 and the forward and reverse primers used to amplify viral RNA were 5′-GAAGAGAAACCCGGAGAAGC-3′ and 5′-CGTAGGCAAACTAAATTCCACAG-3′, respectively. The copy number of the viral RNA was then quantified according to a previously published method^39^ with minor modifications. The reaction was carried out in a QuantStudio 3 Real-time PCR machine (Thermo-Fisher, USA). The copy numbers of the viral RNA estimated by a QuantStudio 3 Real-time PCR machine were normalized by dividing the weight of the lung and reported as the copy numbers of the viral RNA/mg in the lung.

#### Pathological examination

All pigs were necropsied at 7 DPC, PRRSV-induced pneumonia lung lesions were evaluated according to a previously described method^40^. For the macroscopic lung lesion score, the lungs were scored to estimate the percentage of the lung affected by pneumonia. Each lung lobe was assigned a number to reflect the approximate percentage of the volume of the entire lung, and the percentage volume of each lobe was added to the entire lung score (ranging from 0 to 100% of the affected lung).

For the microscopic lung, lung sections were stained for hematoxylin and eosin (H&E) as described previously^41^ and then examined by a blinded observer and given an estimated score according to the severity of interstitial pneumonia. Lung sections were scored according to the severity of interstitial pneumonia as follows; 0 = no microscopic lesions; 1 = mild interstitial pneumonia; 2 = moderate multifocal interstitial pneumonia; 3 = moderate diffuse interstitial pneumonia; 4 = severe interstitial pneumonia. The mean values of microscopic score of each group were calculated.

### Statistical analyses

The data from repeated measurements were expressed as the mean ± SD or SEM and analyzed using analysis of variance (ANOVA) followed by Turkey’s HSD post hoc test by SPSS version 19 (SPSS Inc., IL, USA). The statistical significance was assessed as *p* < 0.05.

## Results

### Formulation

#### Characterization of polylactic acid nanoparticle-encapsulated inactivated PRRSV

**Figure 1:**
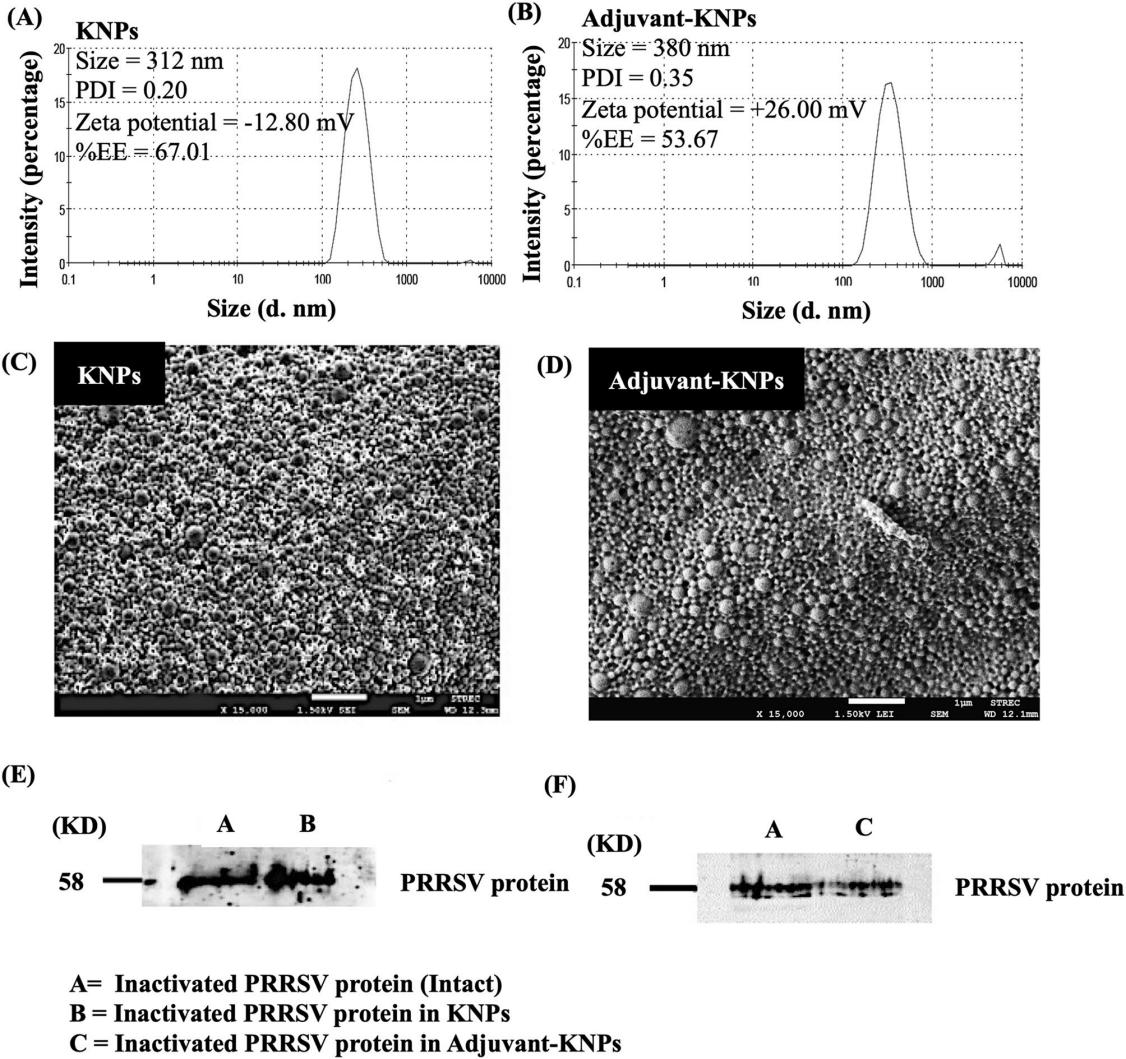
Characteristics of PLA nanoparticles loaded with inactivated PRRSV. Size distribution graph of the (**A**) KNPs and (**B**) adjuvant-KNPs. SEM analysis of the (**C**) KNPs and (**D**) adjuvant-KNPs (1500 × magnification). The scale bar represents 1 μm. Expression of PRRSV protein before and after encapsulation in the (**D**) KNPs and (**E**) adjuvant-KNPs. The existence of PRRSV protein was determined by western blot analysis (full blot see Supplementary Fig. S2). The total proteins in each blot were loaded in equal amounts.

The average size of PLA nanoparticles loaded with inactivated PRRSV (KNPs) was 312 nm, and they had a PDI of 0.20, zeta potential of − 12.80 mV and %EE of 67.01 (Fig. 1A). After coupling the PLA nanoparticles with DDA and LTB (adjuvant-KNPs), the size of the adjuvant-KNPs was increased to 380 nm, with a PDI of 0.35. The zeta potential of the adjuvant-KNPs showed a positive value of 26.00 mV, and the %EE of the adjuvant-KNPs was reduced to 53.67 (Fig. 1B). The SEM images indicated that both nanoparticle systems were spherical in shape with a smooth surface (Fig. 1C,D). The western blot results showed that the PRRSV protein remained intact in both nanoparticle systems (Fig. 1E,F).

### In vitro cell culture studies

#### Cell viability assay

**Figure 2:**
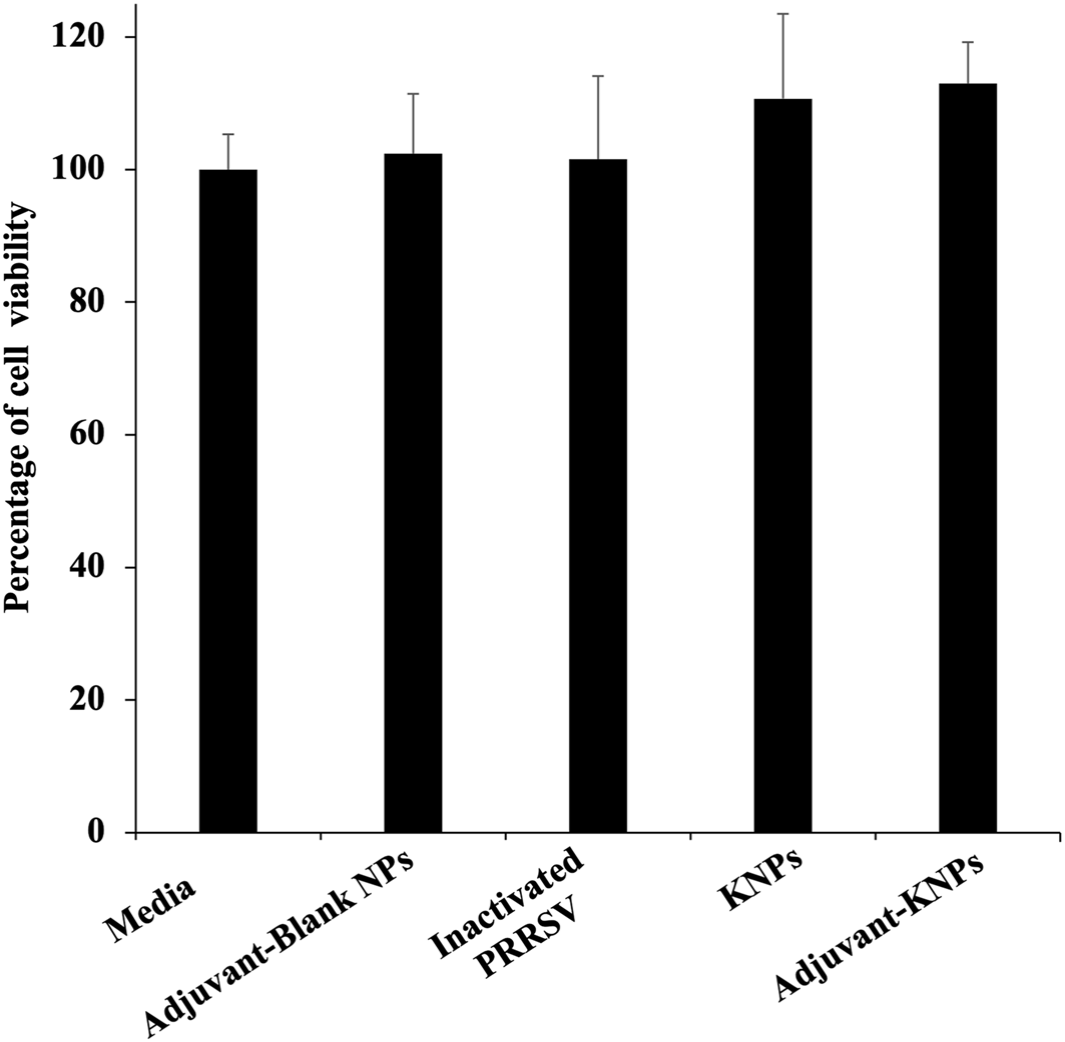
Effects of the various treatments on the viability of PAMs. Cells were treated with inactivated PRRSV, adjuvant-blank NPs, KNPs and adjuvant-KNPs. Cell viability was analyzed using the MTT assay, and each sample was analyzed in triplicate.

To investigate the effect of the PLA-based nanoparticle formulation on cell viability, PAMs (3D4/2 cells) were treated with each nanoparticle formulation and inactivated virus. Cell viability was analyzed using a MTT assay. The PAMs are continuous porcine cell lines developed from alveolar macrophages. The cells replicate upon stimulation and die upon exposure to toxicity. Therefore, they were used for the stimulation and toxicity tests. The results demonstrated that all nanoparticle formulations tested in the study were nontoxic to cells and stimulated the proliferation of 3D4/2 cells. The cell viability was 102.42 ± 9.01%, 101.55 ± 12.57%, 110.71 ± 12.79% and 112.95 ± 6.28% for PAMs treated with adjuvant-blank NPs, inactivated PRRSV, KNPs and adjuvant-KNPs, respectively (Fig. 2).

#### PAMs producing IFN-γ

**Figure 3:**
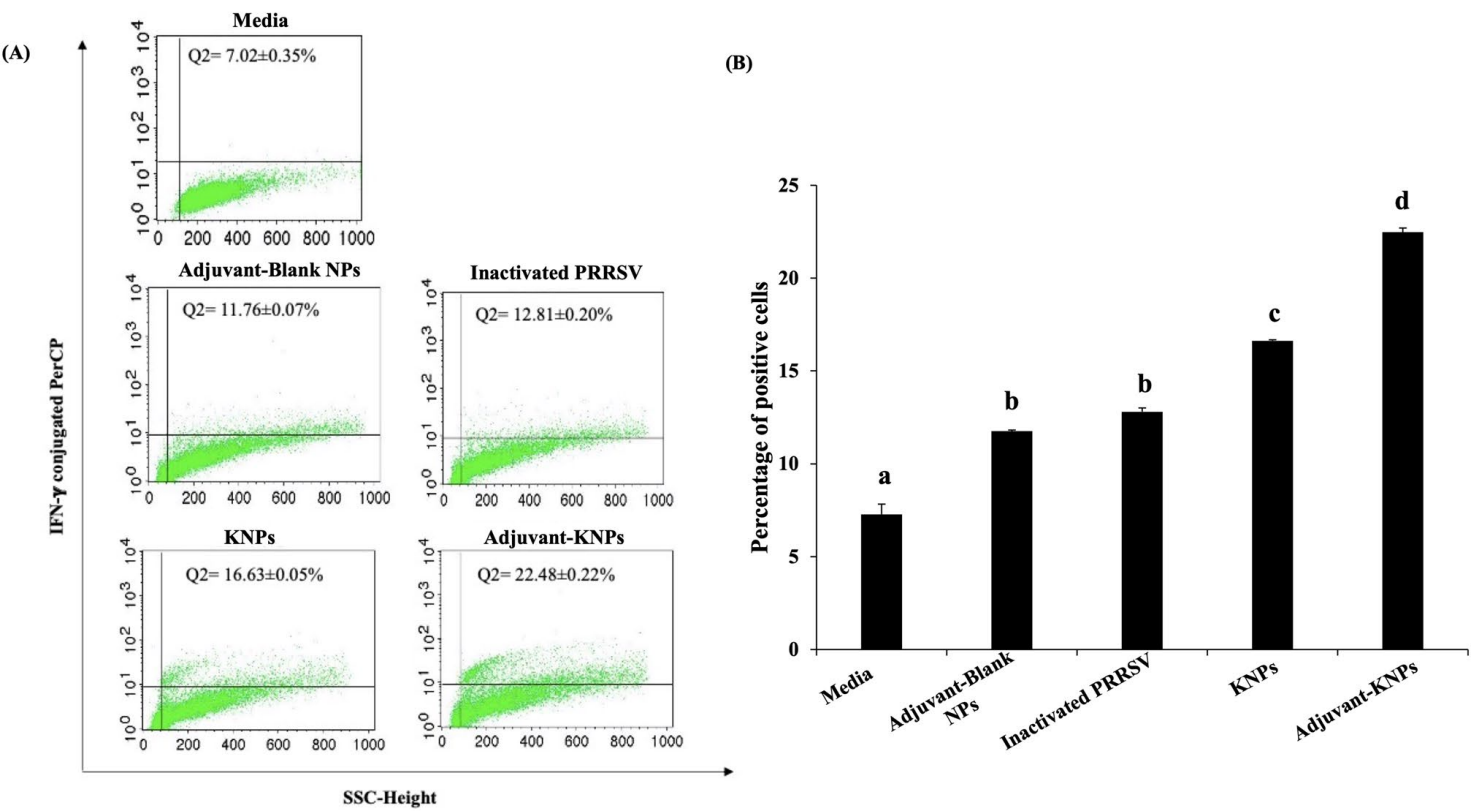
Effects of the various treatments on the production of IFN-γ in PAMs after treatment with adjuvant-blank NPs, inactivated PRRSV, KNPs and adjuvant-KNPs for 24 h. (**A**) Upper right quadrant (Q2) of flow cytometry scatter plots shows the percentage of IFN-γ positive cells. (**B**) The bar graph shows the percentage of IFN-γ positive cells. The data represents the mean ± SD (n = 3). Different lowercase letters indicate significant differences between groups (*p* < 0.05).

To study how PLA nanoparticles help inactivated PRRSV induce interferon gamma production in in vitro cell culture, PAMs (3D4/2 cells) were incubated with inactivated PRRSV, KNPs, adjuvant-KNPs and adjuvant-blank NPs. Following a 48 h incubation, homologous PRRSV was added and incubated for another 48 h. The expression of IFN-γ was then measured by flow cytometry. The results demonstrated that statistically significant differences were not observed in the percentage of IFN-γ-expressing cells between cells treated with inactivated PRRSV and adjuvant-blank NPs (control nanoparticles). Both KNPs and adjuvant-KNPs (LTB-DDA coupled PLA nanoparticles-encapsulating PRRSV) stimulated significant increases in IFN-γ expressing cells in PAMs compared to the control group (*p* < 0.05, Fig. 3A,B). The highest percentage of IFN-γ-expressing cells was detected in PAMs treated with adjuvant-KNPs.

### Animal experiments

#### IFN-γ producing cells

**Figure 4:**
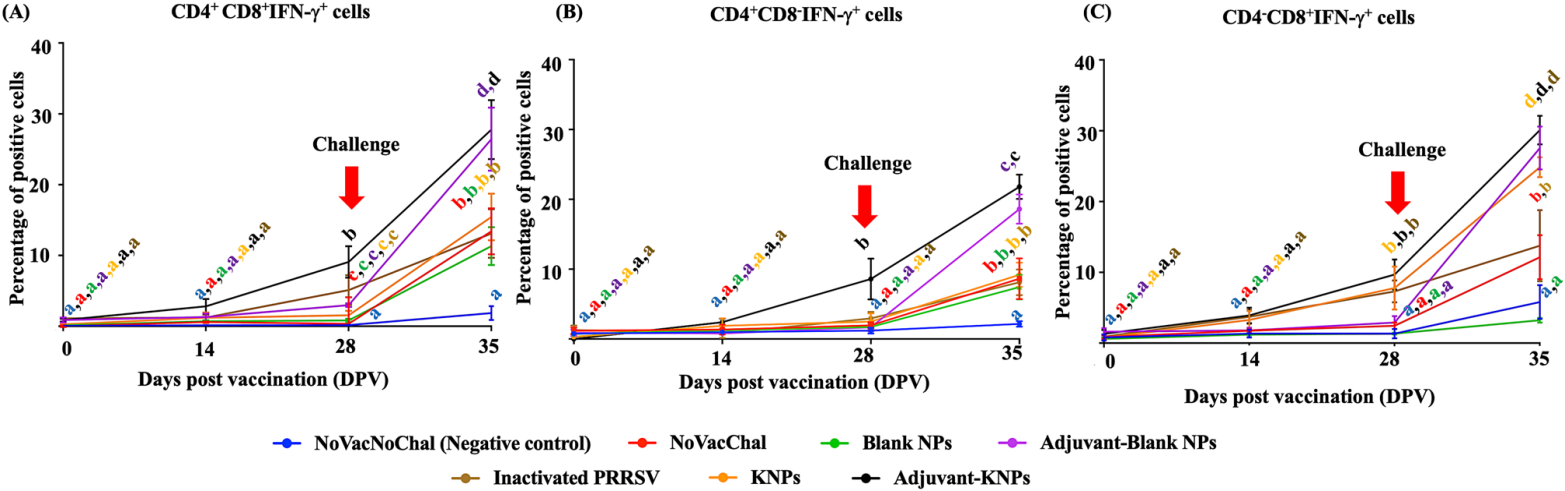
PRRSV-specific lymphocyte populations producing IFN-γ following vaccination. The IFN-γ-producing lymphocyte populations were identified by flow cytometry using cell surfaces and intracellular IFN-γ staining including (**A**) CD4^+^CD8^+^IFN-γ^+^ cells, (**B**) CD4^+^CD8^−^IFN-γ^+^ cells and (C) CD4^-^CD8^+^IFN-γ^+^ cells. The data represents the mean ± SEM. The difference of lowercase letters exhibited significant differences between groups (*p* < 0.05).

Increased numbers of IFN-γ-producing cells in the KNP, adjuvant-KNP and inactivated PRRSV groups were observed as early as 14 DPV. Increased IFN-γ-producing cells in the adjuvant-KNP group trended toward CD4^+^CD8^+^, CD4^+^CD8^−^, and CD4^−^CD8^+^ cells. The number of CD4^+^CD8^+^ and CD4^+^CD8^−^ IFN-γ producing cells, however, was not different from that of the other vaccinated and negative control groups. At 28 DPV, the number of CD4^+^CD8^+^ and CD4^+^CD8^−^ IFN-γ producing cells in the adjuvant-KNP group continued to increase, and the level was significantly higher than that of the other groups. The number of CD4^−^CD8^+^ IFN-γ-producing cells in the KNP, adjuvant-KNP and inactivated PRRSV groups also increased at 28 DPV, and although the number of CD4^−^CD8^+^ IFN-γ-producing cells did not differ between them, the number of cells was significantly higher than that of the other groups. Following challenge at 35 DPV or 7 days post-challenge (DPC), regardless of the lymphocyte populations, the number of IFN-γ-producing cells significantly increased in all vaccinated pigs (Fig. 4A–C).

#### Quantification of porcine IL-10

**Figure 5:**
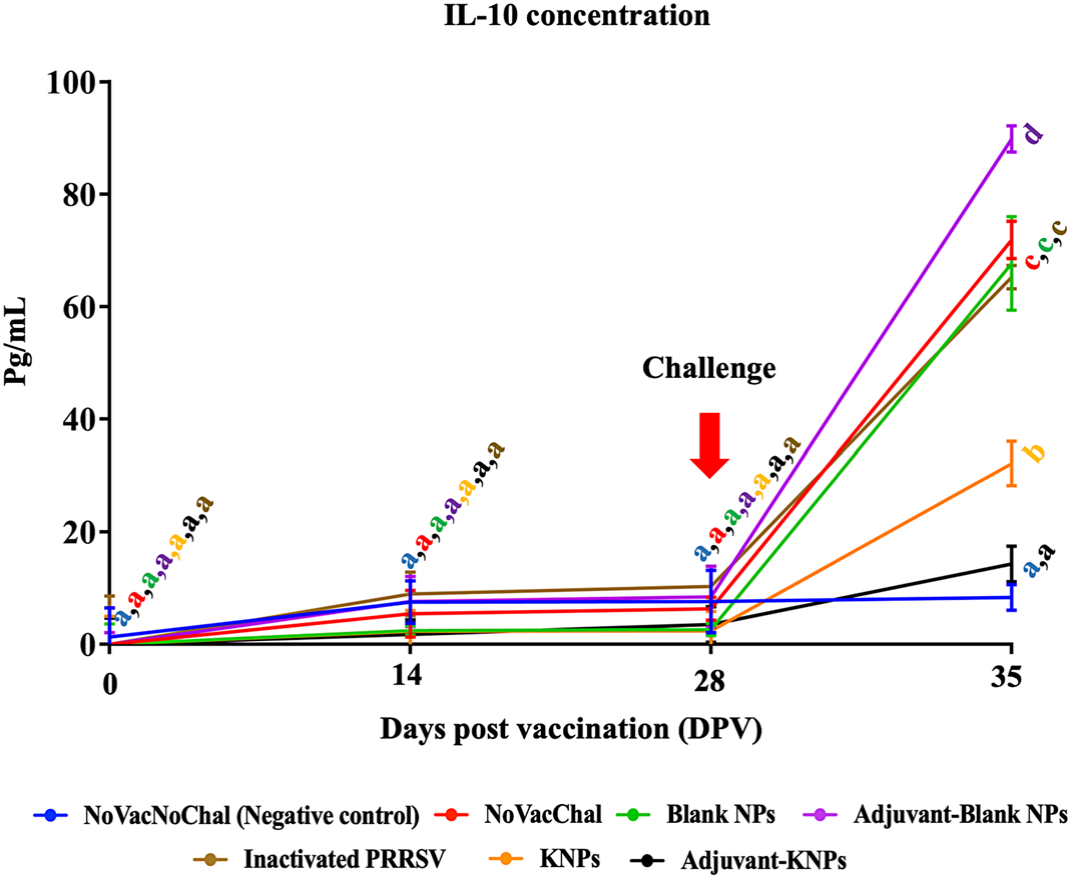
Quantification of porcine IL-10 concentration in the supernatant of stimulated PBMCs. The data represents the mean ± SEM. Different lowercase letters indicate significant differences between groups (*p* < 0.05).

At 14 and 28 DPV, the porcine IL-10 concentration in the supernatant of stimulated PBMCs was relatively low in all experimental groups and showed no significant differences between them (Fig. 5). At 35 DPV or 7 DPC, the IL-10 levels in most of the experimental groups were significantly increased compared to those in the negative group (NoVacNoChal). Interestingly, the IL-10 level of the adjuvant-KNP group was significantly lower at 35 DPV than that of the other experimental groups, and the level of IL-10 was not different compared to that of the negative group.

#### Antibody responses as measured by neutralization assay

**Figure 6:**
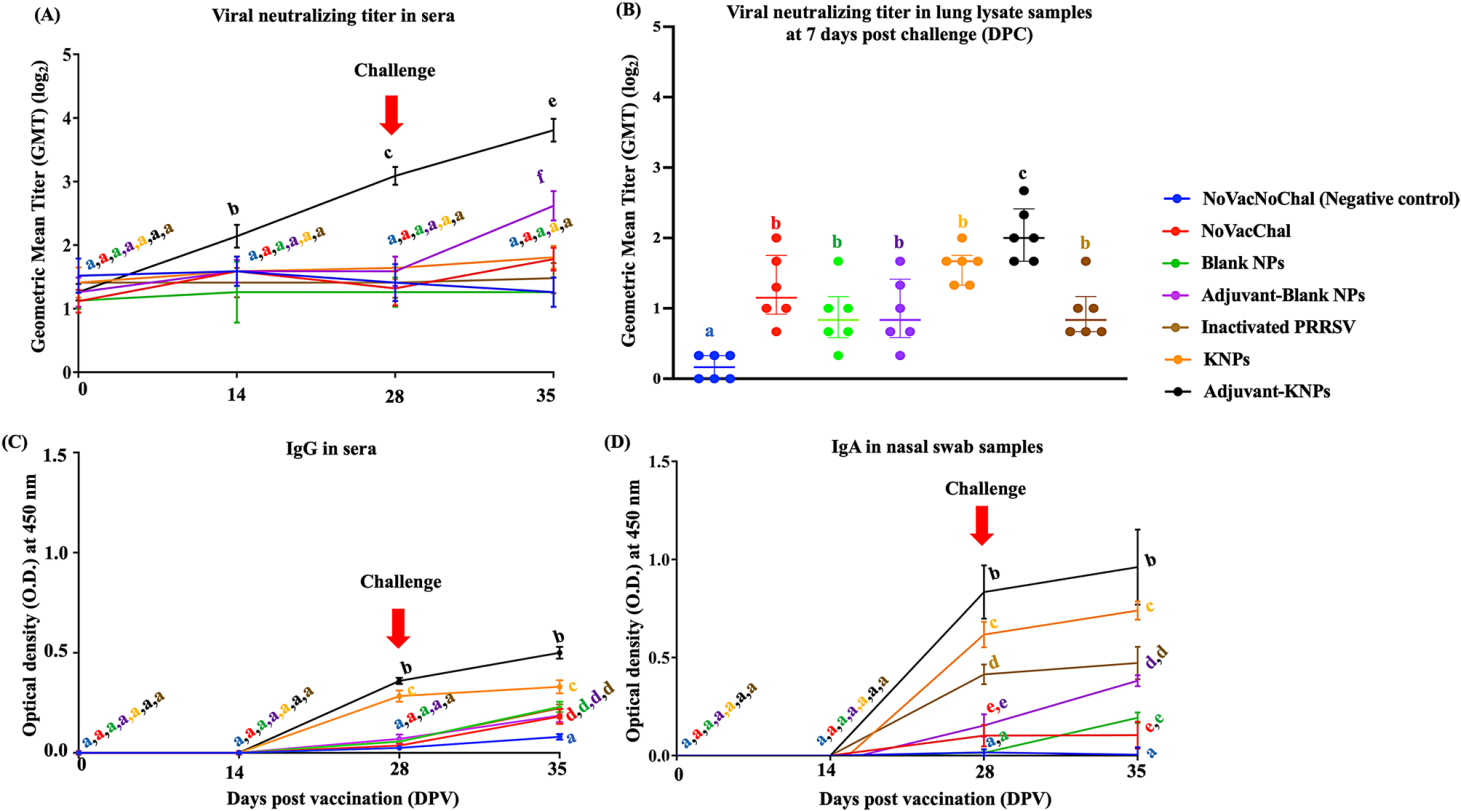
Humoral immune responses in experimental pigs. Analyses were performed to determine (**A**) virus neutralizing titer (VNT) in sera, (**B**) VNT in lung lysate samples, (**C**) anti-PRRSV IgG antibody response in sera, and (**D**) anti-PRRSV IgA antibody response in nasal swab samples. The data represent the mean ± SEM. Different lowercase letters indicate significant differences between groups (*p* < 0.05).

Pigs in negative control (NoVacNoChal), NoVacChal, nanoparticle control (Blank NPs and Adjuvant-Blank NPs), inactivated PRRSV and KNPs groups remained serologically negative throughout the experiment. In contrast, the Adjuvant-KNPs group had a detectable level of VNT at 14 DPV, and the titer gradually increased at 28 and 35 DPV. The VNT at 14, 28 and 35 DPV were significantly higher than that of other groups (*p* < 0.05) (Fig. 6A). A slightly increased in VNT was observed in the Adjuvant-Blank NPs group at 35 DPV. Similar results were observed with the lung lysate samples. The VNT of lung lysate samples in the Adjuvant-KNPs group was highest and the titer was significantly higher compared to the other groups (*p* < 0.05, Fig. 6B) at 7 DPC.

#### Antibody responses as measured by ELISA

Antibody response was not detected in the negative group (NoVacNoChal) throughout the study. At 28 DPV, the Adjuvant-KNPs and KNPs group had detectable levels of IgG in sera and the levels were significantly higher compared with the other groups. The IgG levels in sera of these 2 groups continues to increase at 35 DPV (*p* < 0.05, Fig. 6C) and again the level was significantly highest in the Adjuvant-KNPs group.

In nasal swab samples, the detectable IgA levels were observed in the Adjuvant-KNPs, KNPs and inactivated PRRSV groups as early as 28 DPV and the levels were significantly higher than in that of other groups (*p* < 0.05, Fig. 6D). Both IgG and IgA level of the Adjuvant-KNPs and KNPs groups continued to increase and the levels were significantly higher compared with the other groups at 35 DPV.

#### Quantification of PRRSV RNA in lungs

**Figure 7:**
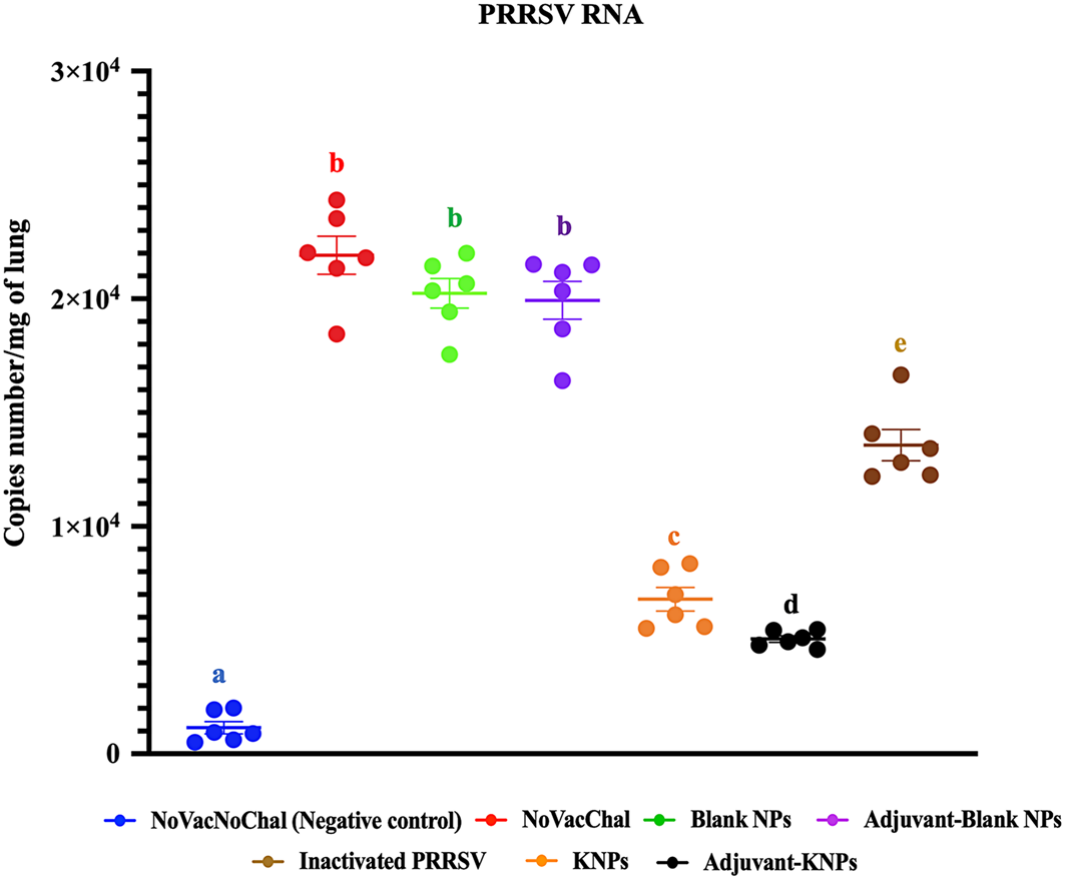
PRRSV RNA copy numbers in lung homogenate samples. Copy numbers were analyzed using qRT–PCR. The data indicate the average RNA copy number per milligram of lung ± SEM (n = 6). Different lowercase letters indicate significant differences between groups (*p* < 0.05).

PRRSV RNA was not detectable in lungs of the negative control group (NoVacNoChal). The viral RNA copy number of the KNPs, Adjuvant-KNPs, and inactivated PRRSV groups were significantly lower compared with the other groups at 7 DPC. No significant differences in viral RNA copy number among the control nanoparticle groups (Blank NPs and Adjuvant-Blank NPs) and NoVacChal groups. Pigs in the Adjuvant-KNPs showed significantly lower viral RNA copy number than in other groups (*p* < 0.05, Fig. 7).

### Pathological examination

#### Macroscopic lung lesion scores

**Figure 8:**
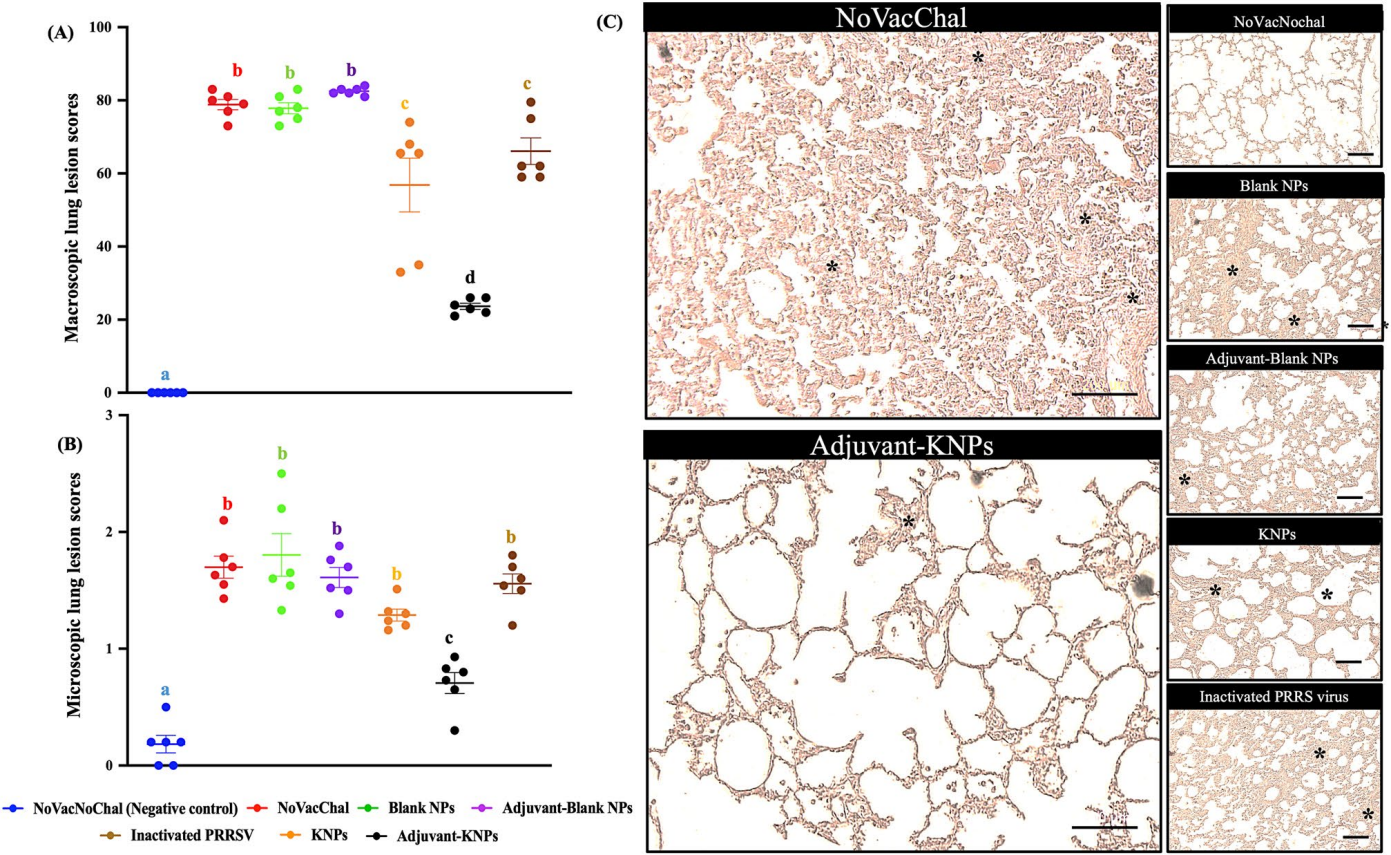
Lung lesion scores at 7 days post-challenge. (**A**) Macroscopic lung lesion scores, (**B**) microscopic lung lesion scores and (**C**) H&E images of microscopic lung lesions from challenge pigs. Different lowercase letters indicate significant differences between groups (*p* < 0.05). *Histopathology of lungs with pneumocyte hyperplasia.

Macroscopic lung lesions were characterized by multifocal, mottled tan areas with irregular and indistinct borders. The macroscopic lung lesion scores are summarized in Table 1. No macroscopic lung lesions were observed in the negative control group (NoVacNoChal). The nonvaccinated, challenged group (NoVacChal) and the control nanoparticle groups (blank NPs and adjuvant-blank NPs) had significantly higher macroscopic lung lesion scores than the other groups, and differences were not observed between them. Among the three vaccinated groups, the adjuvant-KNP group had significantly lower PRRSV-induced pneumonic macroscopic lung scores than the other vaccinated groups (*p* < 0.05, Fig. 8A,C).

**Table 1:** Levels of PRRSV RNA in lungs, and macro- and microscopic lung lesions, lung lesions of intranasally vaccinated pigs following challenged with PRRSV-2 at 7 days post challenge.

Treatments	Descriptions	PRRSV RNA (10^3^ copies/mg)	Macroscopic lung scores	Microscopic lung scores
NoVacNoChal	Negative control	1.27 ± 0.4^a^	0.00 ± 0.0^a^	0.13 ± 0.1^a^
NoVacChal	Challenge control	22.0 ± 0.3^b^	78.8 ± 3.4^b^	1.78 ± 0.3^b^
Blank NPs		20.0 ± 0.7^c^	80.1 ± 1.7^b^	1.83 ± 0.2^b^
Adjuvant-Blank NPs		19.6 ± 1.2^c^	82.5 ± 1.0^b^	1.58 ± 0.5^b^
KNPs		5.81 ± 1.1^d^	56.8 ± 1.7^c^	1.10 ± 0.3^c^
Adjuvant-KNPs		5.03 ± 0.3^e^	23.7 ± 2.1^d^	0.73 ± 0.4^d^
Inactivated PRRSV		7.63 ± 0.4^f^	66.1 ± 8.7^e^	1.63 ± 0.5^b^

Values are present in mean ± SEM. The difference of lowercase letters exhibited significantly differences between groups (*p* < 0.05) in each column.

#### Microscopic lung lesion scores

Microscopic lung lesions associated with PRRSV infection were characterized by thickened alveolar septa with increased numbers of interstitial macrophages and lymphocytes and by type II pneumocyte hyperplasia. The pigs in the negative group had the lowest microscopic lung lesion scores. The microscopic lung lesion scores were consistent with the macroscopic lung lesion scores (Table 1). The nonvaccinated, challenged group (NoVacChal) and the control nanoparticle groups (blank NPs and adjuvant-blank NPs) had the highest microscopic lung lesion scores. Although there was no difference between them, the scores were relatively higher than those of the KNP and inactivated PRRSV groups. The adjuvant-KNP group had a significantly lower microscopic lung lesion score than the other groups (*p* < 0.05) (Fig. 8B,C).

## Discussion

This study was conducted to investigate the induction of systemic and mucosal immune responses and the protective efficacy following the intranasal administration of inactivated PRRSV loaded in PLA-based nanoparticles coupled with LTB and DDA (adjuvant-KNPs). It was demonstrated that pigs intranasally administered adjuvant-KNPs had a strong positive influence on both systemic and mucosal immune responses, as observed by increased VNT and IgG levels in sera and increased IgA levels in the nasal swab samples. A significantly induced cell-mediated immune response in the adjuvant-KNP group was indicated by the increase in IFN-γ-producing lymphocytes and lower level of IL-10 secreted from PBMCs compared to that in the other vaccinated groups. The lowest lung lesion scores and PRRSV RNA copies in the lungs following PRRSV-2 challenge indicated the protective efficacy of the adjuvant-KNP group. The study suggested that when administered intranasally, PLA-based nanoparticles coupled with LTB and DDA are crucial adjuvants to improve the efficacy of inactivated PRRSV in eliciting immune responses and providing protective efficacy.

The findings presented herein suggest the development of intranasal vaccines that can be used for livestock. One of the obstacles for the success of intranasal vaccines is the transport of antigens across M cells to be taken up by immune cells. Our previous in vitro studies indicated that when a cationic lipid DDA is incorporated into the matrix of PLA, it creates positively charged PLA nanoparticles^27^. The positive charge on the surface of nanoparticles is an important key to inducing the initial contact between sialic acid and the heparan sulfate proteoglycan receptor on the surfaces of porcine alveolar macrophages, which facilitates the cellular uptake of antigens loaded within the DDA-PLA nanoparticles via the endocytosis pathway^27,42^. Moreover, the transport of antigens across cultured M cells is also improved following the combination of LTB, a common mucosal adjuvant, with DDA-PLA nanoparticles^27^. LTB is known to bind to the monosialotetrahexosylganglioside (GM1) receptor. The receptor has easy accessibility to M cell areas and therefore can facilitate the transport of vaccine nanoparticles across M cells to underlying immune cells^42,43^. Thus, the improved induction of the immune response observed in pigs vaccinated with LTB-DDA coupled with PLA nanoparticles loaded with inactivated PRRSV could be contributed by increased antigen transportation into M cells and immune cells by LTB coupled with DDA-PLA nanoparticles^27^. Compared with LTB-DDA-coupled PLA nanoparticles, PLA nanoparticles alone (without DDA and LTB) possess a net negative charge on their surface. It is generally accepted that negatively charged nanoparticles are less internalized by cells than positively charged nanoparticles^44^, which might explain why the pigs in the PLA nanoparticles loaded with the inactivated PRRSV group (KNPs) had weaker immune responses than the pigs in the LTB-DDA coupled with PLA nanoparticles loaded with inactivated PRRSV group. Inactivated virus is poorly immunogenic; therefore, unsurprisingly, pigs intranasally vaccinated with inactivated PRRSV alone displayed poor immune induction.

Inactivated PRRSV vaccines induce no detectable level of immune response when intramuscularly administered to PRRSV-free pigs. However, a rapidly increased immune response was observed following intramuscular vaccination with live virus in naïve pigs previously vaccinated with inactivated PRRSV vaccine^45^. The mechanisms underlying this behavior have not been clarified, although they could be associated with memory cells generated following the immune response. Although intramuscular injection in naïve pigs does not lead to detectable levels of immunity, pigs have already been infected by foreign antigens, such as PRRSV. Although the immune response might have been initiated, the level may be below the sensitivity of current assays, such as ELISA. In the present study, similar evidence was observed. Inactivated virus alone when administered intranasally vaccinated induced no detectable level of immune response. However, a significant increase in the immune response was observed following challenge with live virus compared to that in pigs challenged only with inactivated PRRSV. These results suggested that inactivated PRRSV was present and could potentially induce a low level of immune response. To induce a higher immune response, adjuvants are needed. In this study, PLA nanoparticles coupled with DDA and LTB were selected. Encapsulating inactivated PRRSV in PLA nanoparticles is beneficial to protect the antigen from proteolytic degradation, prolong their bioavailability and maintain slow and sustained antigen release, especially in coupling with DDA^27^. After the uptake of nanoparticles in APCs, the nanoparticles can induce the production of various innate cytokines that regulate humoral and cellular immunity^46^. All of these properties facilitated the induction of better immune responses of inactivated PRRSV when loaded in LTB-DDA coupled with PLA nanoparticles. The findings found in the present study are in accordance with earlier studies that reported an increased uptake of inactivated PRRSV in porcine alveolar macrophages with the assistance of PLGA nanoparticles^25,47^. The intranasal vaccine administration of these PLGA nanoparticles resulted in the induction of significantly higher immune responses compared with the intranasal administration of inactivated virus alone, which elicited no response.

Humoral immunity is crucial for controlling PRRSV infection. Neutralizing antibodies contributed mostly to viral clearance by neutralizing viral infection, thereby inhibiting viral replication in the lung and controlling the systemic spread of PRRS virus^48,49^. We found that adjuvant-KNP-vaccinated pigs elicited the highest humoral immunity in both systemic and local responses against homologous PRRSV, as indicated by higher levels of VNT in both the sera and lung lysate samples, IgG in sera, and IgA in nasal swab samples at 14–35 DPV compared to that in the other groups (*p* < 0.05). The significantly high VNT from the serum of pigs vaccinated with adjuvant-KNPs also correlated with the high level of serum IgG from the same group, implying that adjuvant-KNPs have a strong positive influence on humoral responses in systemic and mucosal sites. This was likely due to the great uptake of adjuvant-KNPs in immune cells facilitated by DDA and LTB^27^. Previous studies reported that inactivated PRRSV vaccines elicit a predominantly humoral immune response, as indicated by increased levels of specific PRRSV antibodies^50^ and VNT, and such immunity was augmented by using nanoparticles^25^. Therefore, it is possible to induce high levels of VNT and elicit an effective memory response against PRRSV by using our vaccine.

For the lung lysate samples, we observed high VNT in the adjuvant-KNP-, KNP- and inactivated PRRSV-vaccinated pigs. The adjuvant-KNP group had the highest VNT, and the inactivated PRRSV group had the lowest VNT. In agreement with our studies, commercially available inactivated PRRSV vaccines induced very low VNT^51,52^, and inactivated PRRSV encapsulated in nanoparticles elicited higher VNT in both serum and lung samples than unencapsulated inactivated PRRS virus^52^. Therefore, it is possible that LTB and DDA could play a role in the production of higher neutralizing antibody titers.

Strong cell-mediated immunity is an essential key for protection against PRRSV infection^53^. Significantly increased IFN-γ producing cells, a key moderator of cell-mediated immunity, in the LTB-DDA coupled with PLA nanoparticles loaded with inactivated PRRSV group could indicate a strong stimulation toward a Th1 response, as indicated by the increased number of IFN-γ producing lymphocytes after vaccination. Consistent with our results, previous studies showed that DDA is capable of producing high levels of IFN-γ in CD4^+^CD8^−^ lymphocytes^54,55^. Additionally, LTB could potentially induce the production of IFN-γ in both CD4^+^CD8^−^ and CD4^−^CD8^+^ lymphocytes^56^. Mechanisms involving LTB and DDA and lymphocyte stimulation are not well understood. However, it may involve the ability of LTB to facilitate transcytosis of antigens loaded in nanoparticles across M cells to underlying APCs^57^ and the adjuvant property of DDA. In addition, DDA could be involved in cross-presentation via major histocompatibility complex (MHC) class I molecules by mediating nanoparticles taken up by APCs^50^ and inducing lysosomal escape due to its positively charged nature^58^.

IL-10 is an anti-inflammatory cytokine that maintains the balance of the immune response, allowing effective pathogen clearance with minimal host damage. The expression of IL-10 has been associated with decreases in cell-mediated immune responses against PRRSV by downregulating the expression of proinflammatory cytokines such as IFN-γ^59^. In this study, the induction of IL-10 levels was low in all vaccinated groups. Following challenge, we observed an increase in IL-10 levels in all vaccinated groups except the adjuvant-KNP group, where the IL-10 level showed no significant difference from the negative control group. It has been proposed that IL-10 induction by PRRSV can result in the ineffective induction of an IFN-γ-specific response. The low induction of IL-10 in the adjuvant-KNP group both during vaccination and after challenge indicated that the LTB-DDA coupled with PLA nanoparticle system did not induce a significant IL-10 response to mediate immune dysfunction, as demonstrated by the high number of lymphocytes producing IFN-γ. This finding suggested that LTB-DDA coupled with PLA nanoparticles could be a good candidate for improving the efficacy of inactivated PRRSV vaccines.

For lung pathogens such as PRRSV, preventing initial infection at local sites could contribute to the success of vaccines. IgA in the respiratory tract is mostly responsible for PRRSV clearance at the nasal mucosa, thus helping control primary infection and limiting shedding and lung infection^20,21^. We observed significant differences in IgA levels in nasal swab samples of pigs in the adjuvant-KNP, KNP and inactivated PRRSV groups compared to the other groups. The highest IgA level was found in the adjuvant-KNP group, and the lowest IgA level was detected in the inactivated PRRSV group. This increased IgA level in the nasal swab samples (Fig. 6D) could also be related to the reduction in the PRRSV RNA copy number in the lungs of the challenged pigs (Fig. 7). These data suggested that induction of strong local mucosal immunity is the most important method of clearing detectable replicating challenge PRRSV.

Following challenge, all vaccination groups (KNP, adjuvant-KNP, and inactivated PRRSV groups) were partially protected against PRRSV challenge, as evaluated by the reduction in PRRSV RNA in the lungs and lung lesion scores at 7 DPC. The greatest potential in reducing the copy number of PRRSV RNA in the lungs and lowering lung lesion scores was observed in the adjuvant-KNP group.

The pathological examination of lungs in both macroscopic and microscopic images was typical of those associated with PRRSV infection. Consequently, pathological examination is a critical process for evaluating the efficacy of PRRSV vaccines. The pathology of the lungs was examined at 35 DPV (7 DPC). A previous study indicated that the most extensive and severe lesions were observed at Day 7 after challenge with PRRSV and that resolved lesions were observed at 21 DPC^60^. Our study revealed that the macroscopic lung lesion scores were consistent with the microscopic lung lesion scores and showed similar trends (Fig. 8A,B). The lowest macroscopic lung lesion scores and microscopic lung lesion scores were observed in the adjuvant-KNP group, suggesting the efficacy of this vaccine in protecting against PRRSV infection in the lungs (Fig. 8C). The results suggested that adjuvant-KNPs showed the greatest ability to induce CMI and HRI at both systemic and local sites and provided protective efficacy against homologous PRRSV challenge.

It is noteworthy that heterologous protection against different PRRSV isolates is less reliable, varying from partial to none. Further studies evaluating the protective efficacy of the intranasal PRRSV vaccine against heterologous strains of PRRSV are needed to demonstrate whether intranasal vaccination can broaden the protection. In the present study, mucosal immunity (IgA) in the nasal cavity might be a key issue that plays crucial roles in protecting the mucous membranes against colonization and invasion by PRRSV and preventing the development of potentially damaging immune responses to PRRSV if they reach the pig body.

In summary, this is the first study to illustrate the efficacy of LTB-DDA coupled with PLA nanoparticles loaded with inactivated PRRSV for inducing humoral and cell-mediated anti-PRRSV immune responses, which better improved immune responses and protected PRRSV infection than PLA nanoparticles loaded with inactivated PRRSV and free inactivated PRRSV. Further studies are needed to evaluate the efficacy of LTB-DDA coupled with PLA nanoparticles loaded with inactivated PRRSV compared with commercially available PRRSV vaccines and to investigate anti-PRRSV cross-protective immunity in vaccinated pigs. Our study successfully produced a novel promising vaccine against PRRS administered by nasal vaccination. The novel vaccine may have the potential to control PRRSV outbreaks and reduce economic losses of swine farms.

## Supplementary Information

Supplementary Information.
