# Supplementary material for: Intranasal delivery of inactivated PRRSV loaded cationic nanoparticles coupled with enterotoxin subunit B induces PRRSV-specific immune responses in pigs
Source: Sci Rep. 2022 Mar 8;12:3725. doi: 10.1038/s41598-022-07680-9 (PMC8904483; doi:10.1038/s41598-022-07680-9)
Supplement: Supplementary file 1 — Supplementary Information. [file 41598_2022_7680_MOESM1_ESM.pdf]

Supplementary

Intranasal delivery of inactivated PRRSV loaded cationic nanoparticles coupled with enterotoxin subunit B induces PRRSV-specific immune responses in pigs

Puwich Chaikhumwang[1], Adthakorn Madapong[2], Kepalee Saeng-chuto[2], Dachrit Nilubol[2] and Angkana Tantituvanont[3,*]

[1] Division of Pharmaceutical Sciences, Department of Pharmaceutical Care, Faculty of Pharmaceutical Sciences, University of Phayao, Phayao, 56000 Thailand
[2] Department of Veterinary Microbiology, Faculty of Veterinary Science, Chulalongkorn University, Bangkok, 10330 Thailand
[3] Department of Pharmaceutics and Industrial Pharmacy, Faculty of Pharmaceutical Sciences, Chulalongkorn University, Bangkok, 10330 Thailand

*Corresponding author: Angkana Tantituvanont
Address: Department of Pharmaceutics and Industrial Pharmacy, Faculty of Pharmaceutical Sciences, Chulalongkorn University, Bangkok, 10330 Thailand.
Tel: +66 22 188 402.
Email: tuvanont@gmail.com, angkana.T@pharm.chula.ac.th

Supplementary Figure S1

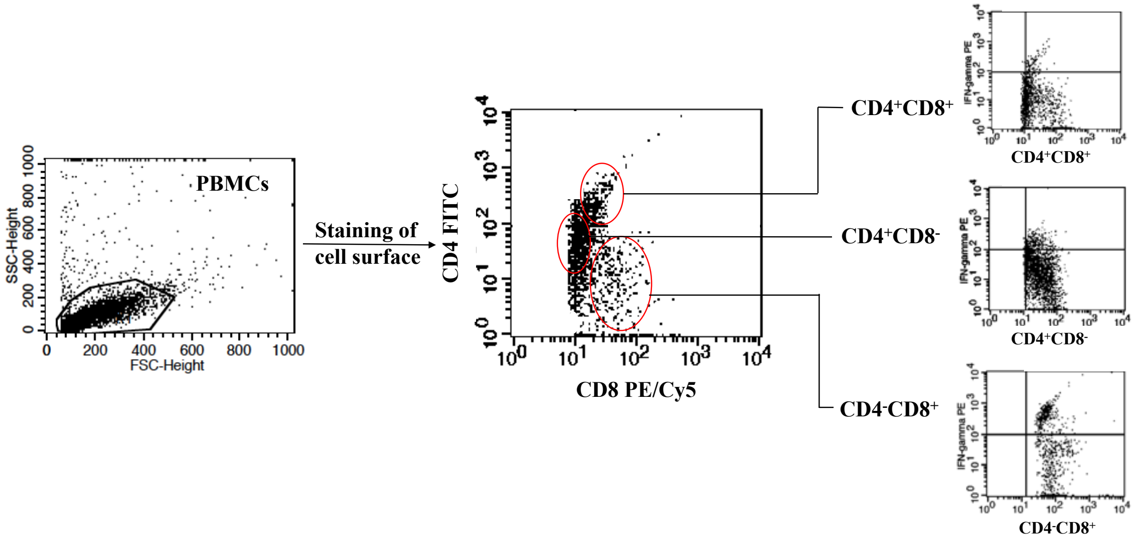

Supplementary Figure S1. The measurement of interferon-γ (IFN-γ)-producing cells using flow-cytometric analysis. The surfaces of the stimulated peripheral blood mononuclear cells (PBMCs) were stained with mouse anti-porcine CD4-FITC-conjugated antibody and mouse anti-porcine CD8-PE/Cy5-conjugated antibody to discriminate between the $CD4^+$ and $CD8^+$ subpopulations. The cells were gated into $CD4^+CD8^+$, $CD4^+CD8^-$ and $CD4^-CD8^+$. After surface staining, the PBMCs were fixed and stained with mouse anti-porcine IFN-γ-PE-conjugated antibody. The stained PBMCs were analyzed for the expression of IFN-γ by flow cytometry.

Supplementary Figure S2

(A)

(B)

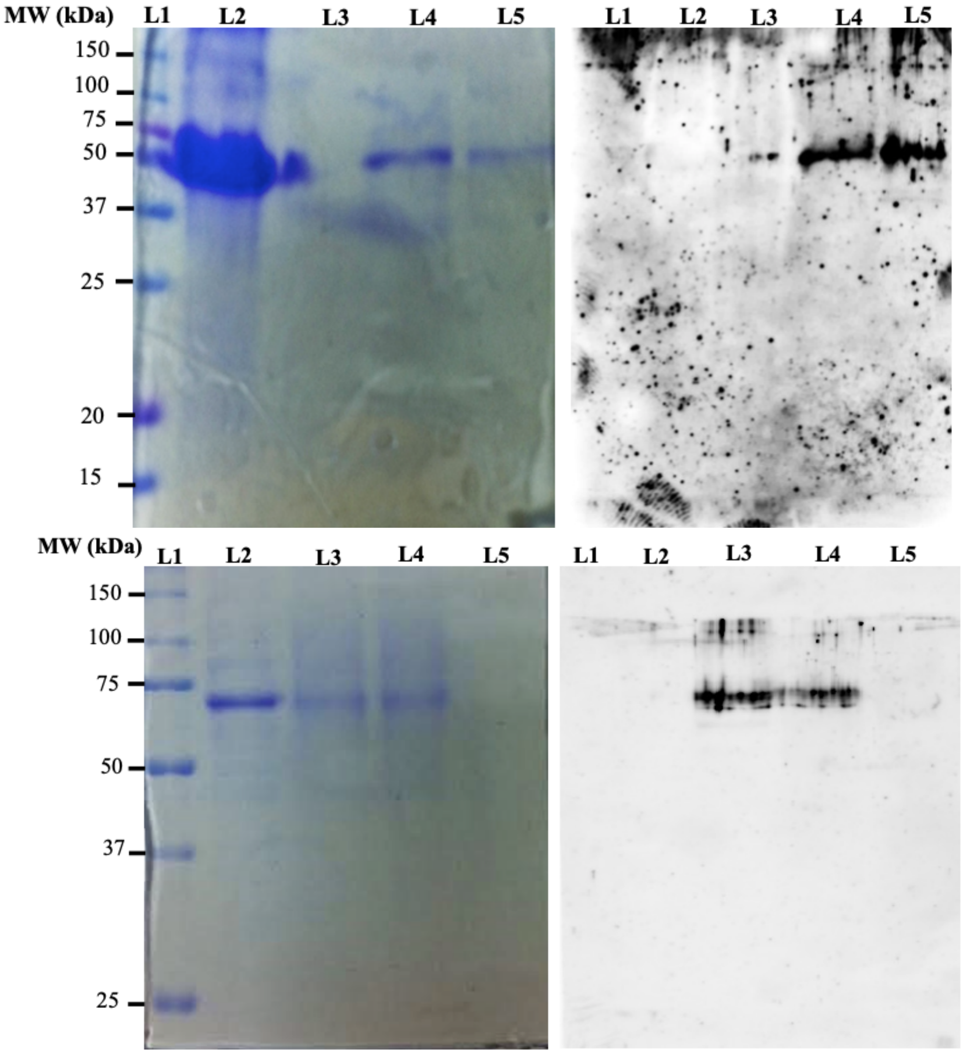

Figure S2A
L1 = ladder
L2 = Bovine serum albumin (BSA)
L3 = Phosphate-buffer saline (PBS)
L4 = Inactivated PRRSV protein (Intact)
L5 = Inactivated PRRSV protein in KNPs

Figure S2B
L1 = ladder
L2 = Bovine serum albumin (BSA)
L3 = Inactivated PRRSV protein (Intact)
L4 = Inactivated PRRSV protein in Adjuvant-KNPs
L5 = Phosphate-buffer saline (PBS)

Supplementary Figure S2. The existence of PRRSV protein was determined by SDS PAGE and Western blot analysis (full-length blot). (A) Inactivated PRRSV protein in KNPs and (B) Inactivated PRRSV protein in Adjuvant-KNPs. The PRRSV proteins in each blot were loaded in equal amounts.
